# Cemented versus uncemented hemiarthroplasty for elderly patients with displaced fracture of the femoral neck: A PRISMA-compliant meta-analysis of randomized controlled trial

**DOI:** 10.1097/MD.0000000000021731

**Published:** 2020-08-14

**Authors:** Binfeng Liu, Ang Li, Jialin Wang, Hongbo Wang, Gongwei Zhai, Haohao Ma, Xiaoyu Lian, Bo Zhang, Liyun Liu, Yanzheng Gao

**Affiliations:** Department of Orthopedics, People's Hospital of Zhengzhou University, Henan Provincial People's Hospital, School of Clinical Medicine, Henan University, Zhengzhou, Henan, China.

**Keywords:** cemented, displaced fracture of the femoral neck, hemiarthroplasty, meta-analysis, uncemented

## Abstract

**Background:**

This meta-analysis was performed to incorporate newly published, high-quality randomized controlled trials (RCTs) to determine the effects of cemented versus uncemented hemiarthroplasty for elderly patients with displaced fracture of the femoral neck.

**Methods:**

The following electronic databases were extensively searched from the inception of the database through December 2018: EMBASE, Medline, the Cochrane Library, and Web of Science. RCTs focusing on the outcomes of cemented and uncemented hemiarthroplasty were reviewed and screened for eligibility. We used the Cochrane Collaboration's Review Manager Software to perform meta-analyses. Two independent reviewers extracted the data and assessed the study quality and bias risk through the Cochrane Collaboration tool. Use fixed effect model or random effect model to pooled data. Cochran's Q statistic was used to evaluate heterogeneity, and I^2^ statistic was used to quantify heterogeneity.

**Results:**

Fifteen RCTs were enrolled (n = 3790) (uncemented hemiarthroplasty group = 1015; cemented hemiarthroplasty group = 1037) (mean age ranged from 70–85.3 years; all patients > 65 years). The meta-analysis showed that cemented hemiarthroplasty has a longer operating time (weighted mean difference, 8.03; 95% confidence interval (CI) 4.83–11.23; *P* < .00001), less pain (odds ratio, 0.48; 95% CI 4.83–11.23; *P* = .02), lower mortality 1-year (odds ratio, 0.78; 95% CI 0.62–0.98; *P* = .03) and fewer implant-related complications (odds ratio, 0.20; 95% CI 0.13–0.30; *P* < .00001) than Uncemented hemiarthroplasty. However, there are still some limitations in our study, such as the uniformity of the surgery administration programme and rehabilitation scheme, and the small sample size of the included studies.

**Conclusions:**

Cemented hemiarthroplasty for elderly patients with displaced fracture of femoral neck may acquire better functional results.

## 1 Introduction

Femoral neck fracture is a common and costly health problem worldwide. With increases in the ageing population and average life expectancy, the frequency of these fractures is steadily increasing.^[1,2]^ This problem is expected to worsen. Hemiarthroplasty is the most common treatment for displaced fractures of the femoral neck in the elderly individuals and is associated with better functional outcome and fewer reoperations than internal fixation.^[3]^ There are 2 different methods for hemiarthroplasty: fixation with bone cement or press-fit without cement.

Many studies, systematic reviews and meta-analyses^[4–7]^ have suggested that cemented hemiarthroplasty can be achieved with less pain by providing an immediate strong interlock between the prosthesis and the periprosthetic bone tissue. Cement fixation can decrease postoperative complications related to late mobilization, such as pneumonia or urinary tract infection, compared with uncemented fixation. However, other studies favor uncemented prostheses as the operative time, blood loss and incidence of perioperative mortality are less.^[8]^ As there is still a dispute about which treatment is more suitable for elderly patients with displaced fracture of femoral neck, we need critical evidence to provide guidance for clinical treatment.

Until recently, few systematic reviews and meta-analyses comparing cemented hemiarthroplasty with uncemented hemiarthroplasty had been published. However, several new randomized controlled trials (RCTs) have been published in recent years. The purpose of this meta-analysis was to include newly published high-quality RCTs to compare the clinical outcome of cemented and uncemented hemiarthroplasty for the treatment for elderly patients with displaced fracture of femoral neck in order to provide the best clinical evidence to provide guidance for clinical treatment.

## 2 Materials and methods

### 2.1 Literature search

The following electronic databases were extensively searched independently by 2 investigators from the inception of the database through December 2018: EMBASE, Medline, the Cochrane Library, and Web of Science. The search strategy was created with the assistance of a librarian using a combination of terms including hemiarthroplasty, femoral neck fracture, hip, hip fracture, bone cement, bone cements, cemented, uncemented, cementless, RCT, prospective, meta, review, and random. We limited searches to RCTs, systematic reviews, and meta-analyses and imposed no language or other limitations. Manual searches of relevant trials, reviews, and related articles were also performed. When possible, authors were contacted to obtain missing information.

### 2.2 Inclusion and exclusion criteria

To be included in this analysis, trials had to fulfil the following inclusion criteria:

(1) RCTs and
(2) studies comparing the outcome of cemented and uncemented hemiarthroplasty; The exclusion criteria included the following:
(3) patients with a previous fracture of the same hip or with a pathological fracture;
(4) case reports, editorials, experimental studies, conference articles, non-English studies, and other studies that failed to report the outcome of interest;
(5) repeated studies and data; and
(6) Articles that did not report any treatment results in the cemented or uncemented groups; 2 authors independently assessed the articles for compliance with the inclusion criteria, and disagreement was followed by discussion until consensus was reached.

### 2.3 Selection of the literature

After removing duplicates, the titles and abstracts were scanned by 2 independent investigators according to predefined selection criteria and potentially relevant RCTs were selected. Hard copies of all relevant articles were retrieved and read in full for further identification. The relevant data were extracted by adapting a predetermined standardized procedure, which involved first authors, year of publication, country, participant demographic characteristics, and treatment regime for each group. Disagreements regarding studies to be included and data abstraction were resolved by consensus or discussion with a 3rd author.

### 2.4 Quality assessment

The Cochrane collaboration's tool for assessing the risk of bias was used to evaluate the methodological quality of the included trials. This tool focuses on the internal validity of the trial and assesses the risk of possible bias in different phases of the trial. The items in the tool are as follows: random sequence generation, allocation concealment, blinding of outcome assessment, blinding of participants and personnel, incomplete outcome data, selective reporting, and other bias. Each item was classified according to a high, low, or unclear risk of bias that is represented as high (H), low (L), and unclear (U), respectively. All of the assessments were conducted by 2 independent reviewers (LBF, LA). Any controversies were settled by consensus or discussion with a 3rd author (WHB).

### 2.5 Data extraction

All data were extracted independently by 2 reviewers. The following data were extracted: mortality, blood loss, operation time, length of hospital stay, residual pain, reoperation rate, complications, and functional outcomes. When only the survival curve was available, mortality was estimated. A consensus method was used to resolve disagreements, and a 3rd reviewer was consulted when disagreements persisted. To understand the baseline of each included study, we extracted data from trials that included the following information: number of patients enrolled, characteristics of participants, male/female ratio, and follow-up time.

### 2.6 Statistical analysis

The Cochrane Collaboration Review Manager Software Package (Rev Man Version 5.3) was used to perform the meta-analyses. The overall effect size of each anesthetic was calculated as the weighted average of the inverse variance for the study-specific estimates. For dichotomous variables, odds ratios (ORs) with the corresponding 95% confidence intervals (CIs) were calculated, and the weighted mean difference (WMD) was used to estimate numerical variables. Heterogeneity was evaluated with the χ^2^ distribution test and Higgins I^2^ index. They were synthesized results was done by pooling the data and using a fixed effects model meta-analysis. However, if the I^2^ indicated moderate or high heterogeneity (i.e., I^2^ above 50%), a random effect model was selected for analysis. As defined by Higgins et al,^[9]^ heterogeneity was tested by Cochran's Q. If essential, subgroup analysis was conducted to identify and explain the heterogeneity, stratifying the data according to different time periods. When only the median, the minimum, the maximum, or the 25th and 75th percentiles were available, the sample mean and standard deviation were estimated

### 2.7 Ethical statement

As all analyses were conducted with data from previously published studies, ethical approval was not necessary.

## 3 Results

### 3.1 Description of studies

**Figure 1:**
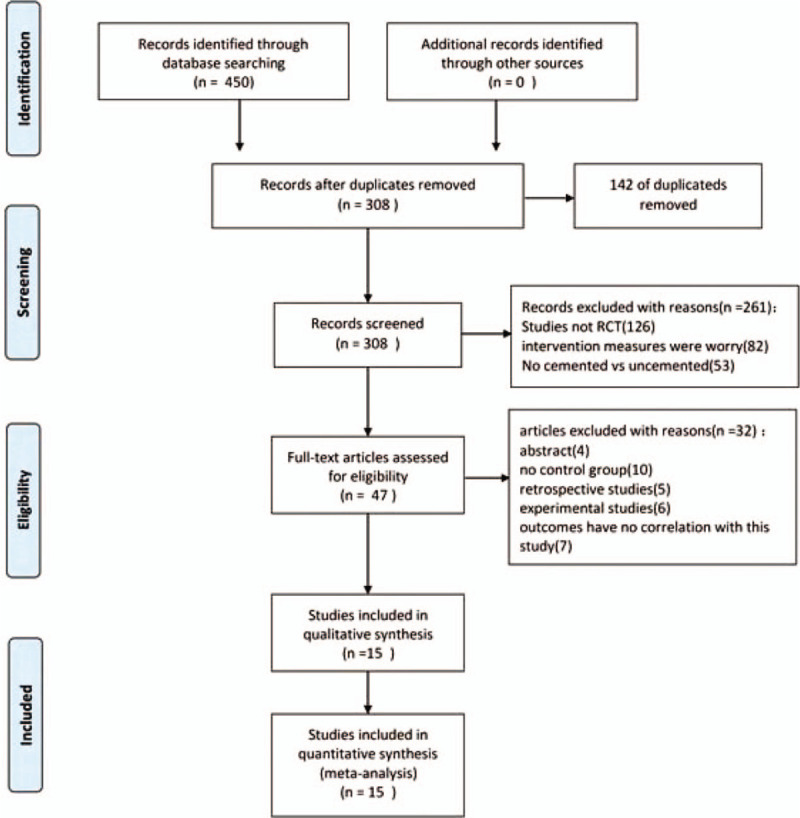
Flow diagram of the studies included.

Figure 1 presents a flowchart describing the process by which we screened and selected trials. The initial literature search yielded 450 articles in all. According to the inclusion and exclusion criteria, duplicate checking and title and abstract screening resulted in 47 publications. One study was published twice with a different length of follow up.^[10,11]^ We only used chose the most recent study.^[11]^ Consequently, 15 studies^[11–25]^ were analyzed in the meta-analysis. All selected studies in our meta-analysis were published between 1977 and 2018 and included 2052 patients: 1015 in the uncemented hemiarthroplasty group and 1037 in the cemented hemiarthroplasty group. The average reported age of the patients ranged from 70 to 85.3 years. Every patient in the included study had a fracture of the femoral neck, and the included study compared the outcomes of cemented and uncemented hemiarthroplasty. More detailed characteristics of the eligible trials are shown in Table 1.

**Table 1:**
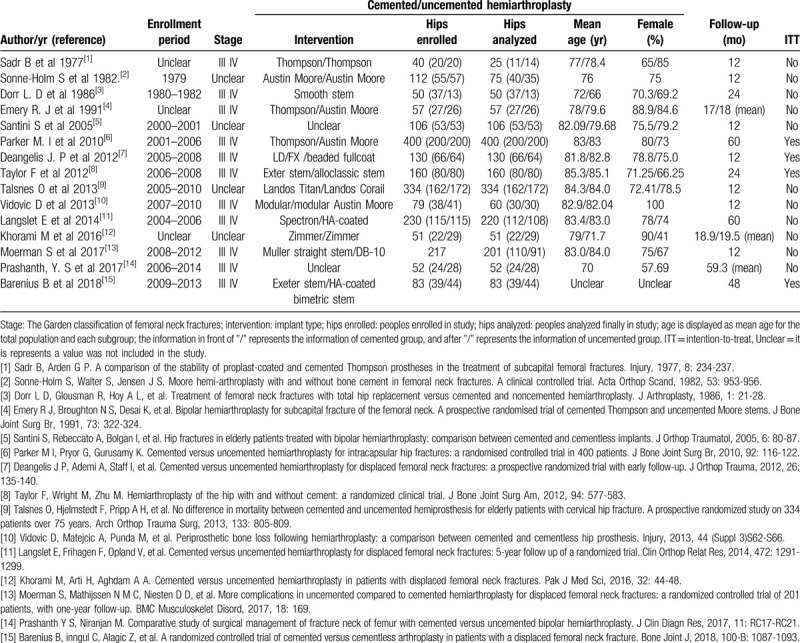
Characteristics of the studies included in the meta-analysis.

### 3.2 Risk of bias

**Figure 2:**
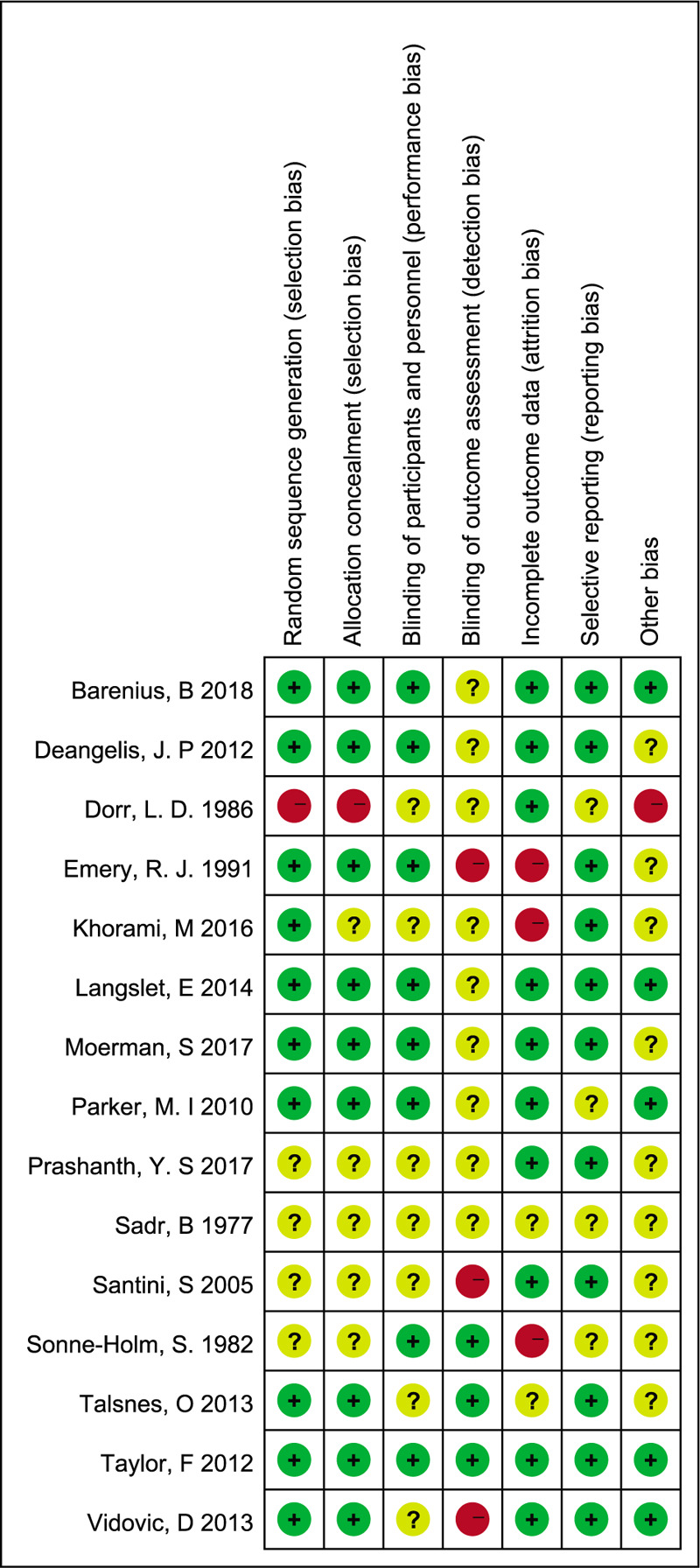
Risk of bias summary of randomized controlled trials.

**Figure 3:**
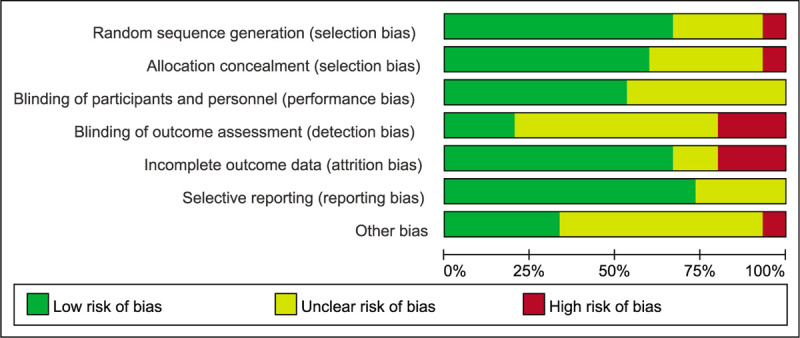
Risk of bias graph of randomized controlled trials.

Overall, the methodological quality of all eligible trials indicated a low risk of bias. Based on the Cochrane Collaboration's recommendations, detailed methods for the random sequence generation were reported in 10 RCTs,^[11,15,17–23,25]^ and 1 study randomized patients based on the patient's hospital number (odd or even),^[14]^ which has a high risk of bias. In total, there were 9 RCTs^[11,15,17–21,23,25]^ with adequate concealment of allocation. The participants and personnel were blinded in 8 studies.^[11,13,15,17–19,23,25]^ Outcome assessors were blinded in 3 studies^[13,19,20]^ while 3 studies^[15,16,21]^ did not have a blind evaluation of the outcomes. The other 9 studies described the blinding procedures unclearly. A more detailed description of the risk of bias and methodological quality of the eligible studies is illustrated in Figures 2 and 3.

### 3.3 Length of hospital stay

**Figure 4:**
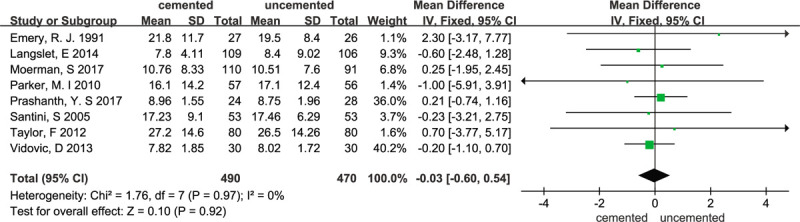
Forest plot for hospital stay.

Eight studies^[11,15–17,19,21,23,24]^ compared the length of hospital stay in this meta-analysis. However, there was no significant difference the between cement group and the non-cement group in these studies. (n = 960; WMD,–0.03; 95% CI,–0.60 to 0.54; Heterogeneity: χ^2^ = 1.76; *P* = .97; I^2^ = 0%). The forest plot is illustrated in Figure 4.

### 3.4 Operation time

**Figure 5:**
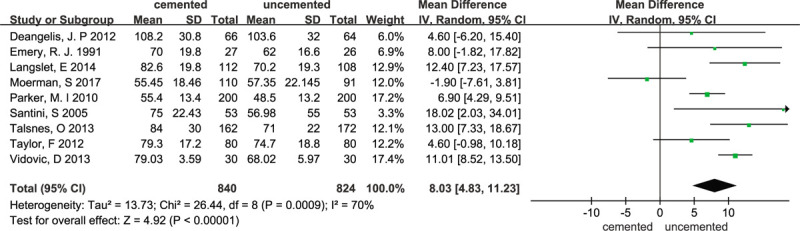
Forest plot for operation time.

**Figure 6:**
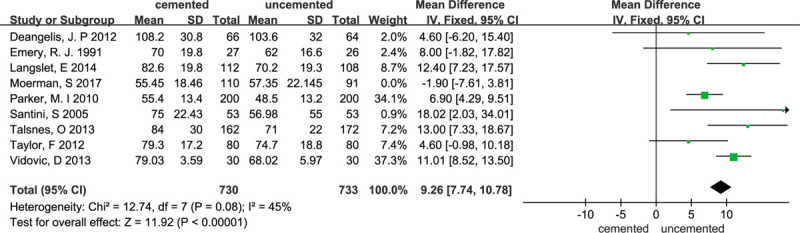
Forest plot for sensitivity analysis of operation time.

A total of 9 trials^[11,15–21,23]^ reported the operation time. The random-effects meta-analysis of all 9 trials showed an increased time of surgery for cemented hemiarthroplasty in comparison with uncemented hemiarthroplasty, with a pooled WMD of 8.03 (95% CI 4.83–11.23). The results were statistically significant (*P* < .00001). Evidence showed that the heterogeneity was high (χ^2^ = 26.44; I^2^ = 70%; *P* = .0009) and the results are presented in Figure 5. Therefore, we performed a sensitivity analysis. After the sensitivity analysis, 1 RCT^[23]^ was excluded and the results are presented in Figure 6. The sensitivity analysis was consistent with our previous analysis (WMD = 9.26, 95% CI 7.74–10.78; *P* < .00001; fixed-effects model) with low heterogeneity (χ^2^ = 12.74; I^2^ = 45%;*P* = .08).

### 3.5 Reoperation rate

**Figure 7:**
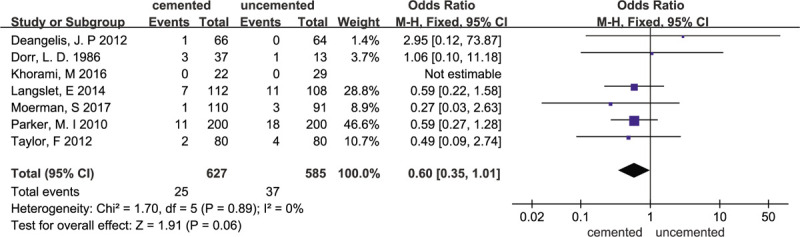
Forest plot for reoperation rates.

Seven studies included data on the reoperation rates reported in those studies.^[11,14,17–19,22,23]^ In total, 25 patients from the cemented group of 627 patients and 37 patients from the uncemented group of 585 patients underwent revision surgery. In 1 trial,^[22]^ no patients underwent revision surgery. The fixed-effects meta-analysis of the 7 trials showed that there was no significant difference in the reoperation rate between the cemented group and the uncemented group. The odds ratio of reoperation for any reason was 0.60 (95% CI 0.35–1.01; *P* = .06), and there was no heterogeneity (χ^2^ = 1.70; I^2^ = 0%; *P* = .89). The forest plot is illustrated in Figure 7.

### 3.6 Residual pain

**Figure 8:**
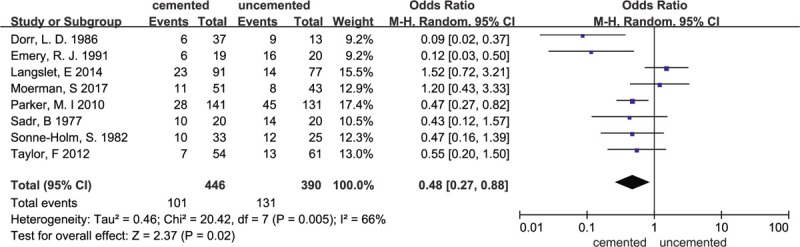
Forest plot for residual pain.

Overall 8 studies^[11–15,17,19,23]^ reported residual pain. Five studies^[11–13,19,23]^ showed no significant difference between the cemented groups and uncemented groups. However, the random-effects meta-analysis of all 8 trials revealed that the cemented groups were associated with less pain (OR = 0.48; 95% CI 0.27–0.88; *P* = .02; random-effects model) compared with the uncemented groups. (Heterogeneity: χ^2^ = 20.42; I^2^ = 66%; *P* = .005). The results are presented in Figure 8.

### 3.7 Blood loss

**Figure 9:**
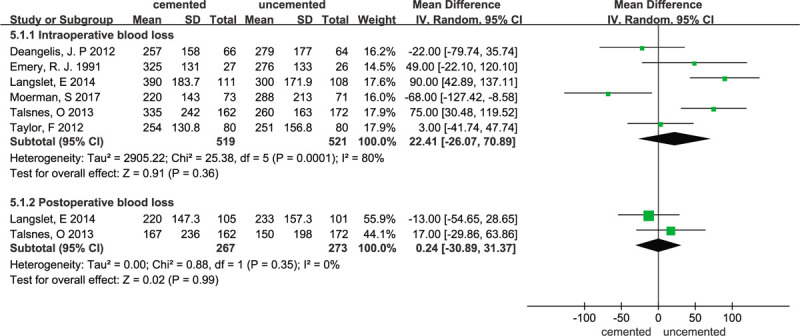
Forest plot for blood loss.

**Figure 10:**
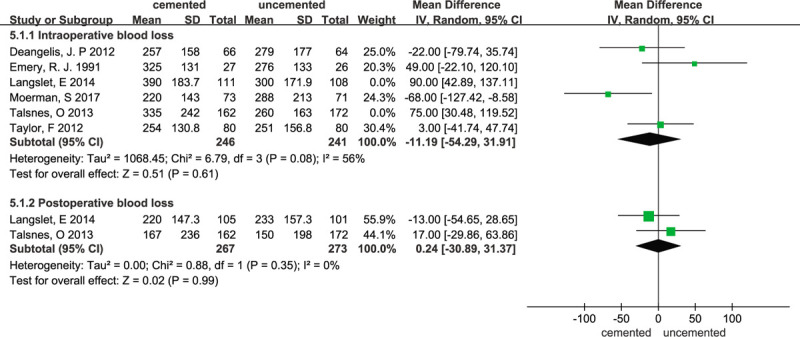
Forest plot for sensitivity analysis of intraoperative blood loss.

Data regarding blood loss were reported in 6 studies.^[11,15,18–20,23]^ All 6 studies reported intraoperative blood loss and 2 studies^[11,20]^ reported postoperative blood loss. The random-effects meta-analysis showed no significant difference in intraoperative blood loss between the 2 groups, with a pooled WMD of 22.41 (95% CI -26.07–70.89; *P* = .36). The forest plot is presented in Figure 9. With respect to the large statistical heterogeneity, the I^2^ value was 80%. To compare the difference and evaluate the sensitivity of the meta-analyses, a sensitivity analysis was performed to evaluate the stability of the meta-analysis. When 2 studies^[11,20]^ were excluded from the meta-analysis, the I^2^ dropped to 56% and the sensitivity analysis is consistent with our previous analysis (WMD = –11.19; 95% CI –54.29 to 31.91 *P* = .61; χ^2^ = 6.79; I^2^ = 56%;random-effects model). The sensitivity analysis are illustrated in Figure 10. The forest plot is presented in Figure 10. The random-effects meta-analysis showed no significant difference in postoperative blood loss between the 2 groups, with a pooled WMD of 0.24 (95% CI –30.89 to 31.37; *P* = .99) and no heterogeneity (χ^2^ = 0.88; I^2^ = 0%; *P* = .35). The forest plot is presented in Figure 9.

### 3.8 Mortality

**Figure 11:**
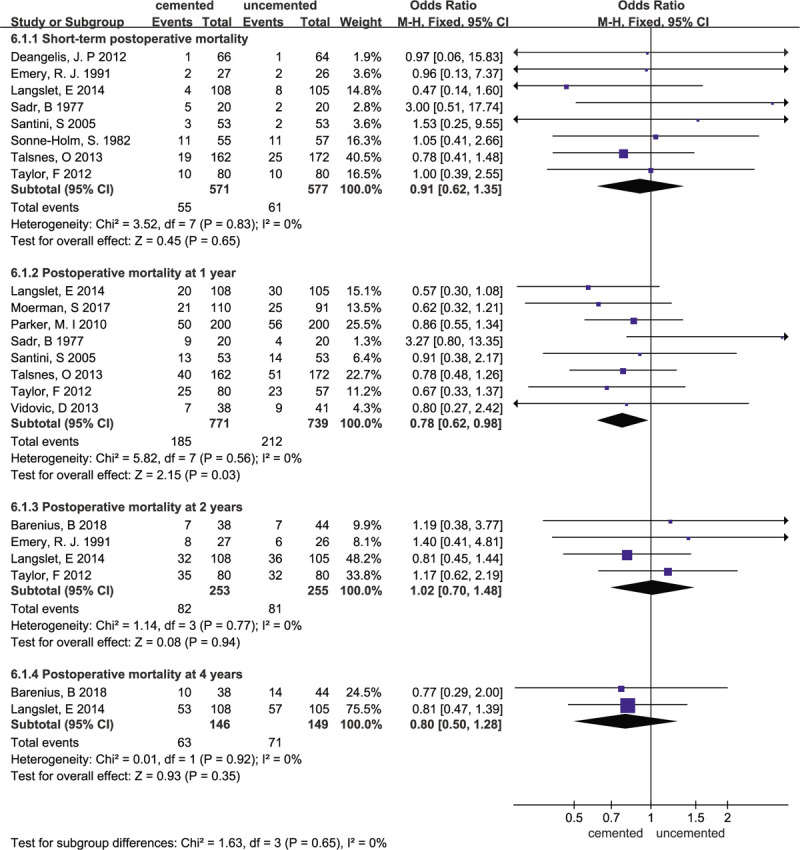
Forest plot for mortality.

Twelve studies^[11–13,15–21,23,25]^ reported mortality at different times. There were no significant differences in short term postoperative mortality between the 2 groups (OR = 0.91; 95% CI 0.62–1.35; *P* = .65; heterogeneity: χ^2^ = 3.52; I^2^ = 0%; *P* = .83). Additionally, no significant differences were detected between the 2 groups for mortality at 2 years (OR = 1.02; 95% CI 0.70–1.48; *P* = .94; heterogeneity: χ^2^ = 1.14; I^2^ = 0%; *P* = .77,) or 4 years (OR = 0.80; 95% CI 0.50–1.28; *P* = .35; heterogeneity: χ^2^ = 0.01; I^2^ = 0%; *P* = .93). However, fixed-effect meta-analysis of 8 trials showed that the mortality at 1 year in the cemented group was lower than that in the uncemented group. (OR = 0.78; 95% CI 0.62–0.98; *P* = .03; heterogeneity: χ^2^ = 5.82; I^2^ = 0%; *P* = .56). Forest plots for mortality at different times are presented in Figure 11.

### 3.9 Harris hip score (HHS)

**Figure 12:**
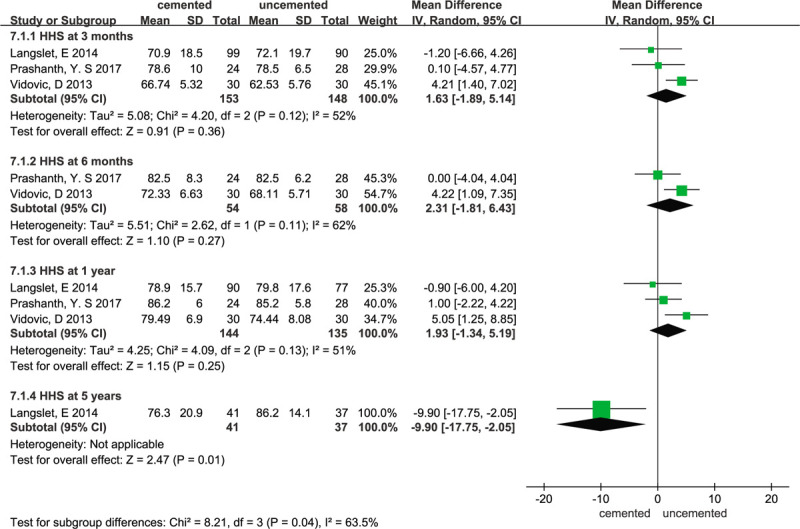
Forest plot for Harris hip score.

Three studies^[11,21,24]^ reported the HHS at different times, such as at 3 months, 6 months, 1 year or 5 years. The random-effect meta-analysis of 3 trials showed no significant difference in HHS at 3 months (WMD = 1.63; 95% CI –-1.89 to 5.14; *P* = .36; heterogeneity: χ^2^ = 4.20; I^2^ = 52%; *P* = .12), 6 months (WMD = 2.31; 95% CI –1.81 to 6.43; *P* = .27; heterogeneity: χ^2^ = 2.62; I^2^ = 62%; *P* = .11), or 1 year (WMD = 1.93; 95% CI –1.34 to 5.19; *P* = .25; heterogeneity: χ^2^ = 4.09; I^2^ = 51%; *P* = .13). However, Langslet et al^[11]^ showed that the HHS at 5 years in the cemented group was lower than that in the uncemented group (WMD = -9.90; 95% CI –17.75 to –2.05; *P* = .01). Forest plots for HHS at different times are presented in Figure 12.

### 3.10 Complication

**Figure 13:**
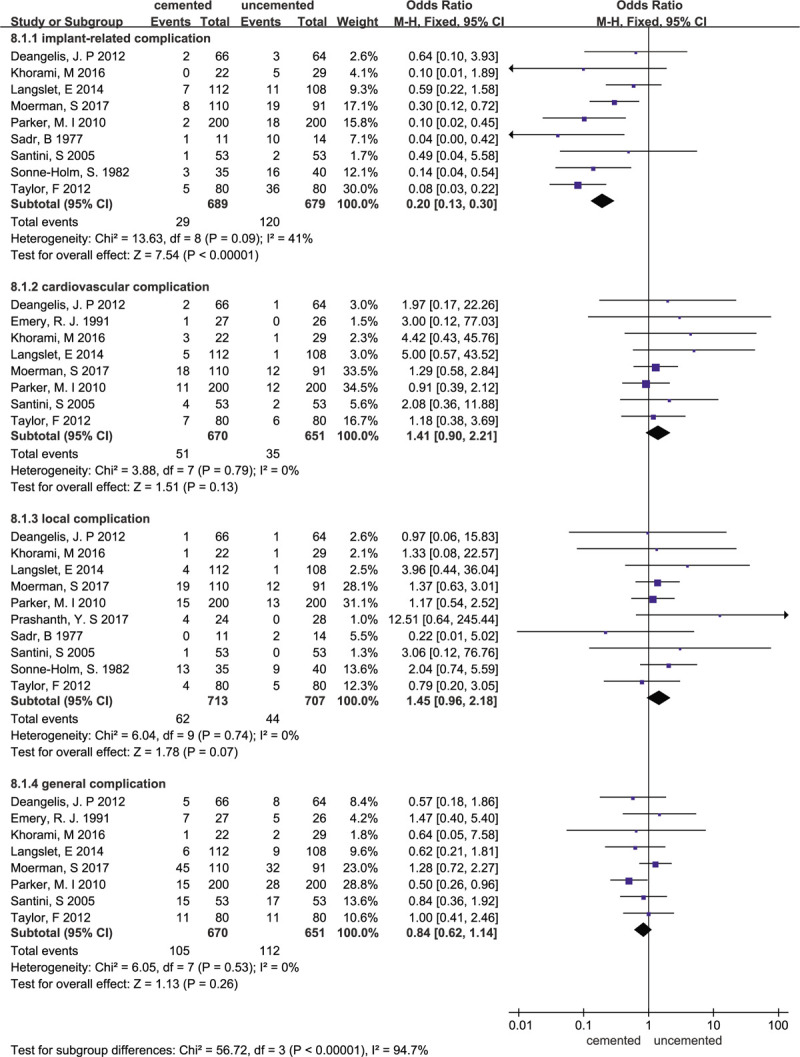
Forest plot for complication.

Eleven studies reported complications. Our findings show that significantly fewer implant-related complications occurred in the cemented group than in the uncemented group (OR = 0.20, 95% CI 0.13–0.30, *P* < .00001), with small heterogeneity (χ^2^ = 13.63; I^2^ = 41%, *P* = .09). However, there was no significant difference between the cemented group and uncemented group in terms of cardiovascular complications (OR = 1.41, 95% CI 0.90–2.21, *P* = 0.13, χ^2^ = 3.88; I^2^ = 0%, *P* = .79), local complications (OR = 1.45, 95% CI 0.96–2.18, *P* = .07, χ^2^ = 6.04; I^2^ = 0%, *P* = .74) and general complications (OR = 0.84, 95% CI 0.62–1.14, *P* = .26, χ^2^ = 6.05; I^2^ = 0%, *P* = .53). The forest plot is presented in Figure 13.

## 4 Discussion

Our study showed that there were no significant differences in the length of hospital stay between the 2 groups, which was consistent with 2 previous meta-analyses.^[26,27]^ We also found there is no statistically significant difference in residual pain, which was different from 2 systematic reviews.^[5,28]^ On the one hand, Xiang ping Luo et al^[5]^ and Azegami et al^[28]^ suggested that the residual pain in the cemented group was lower than that in the uncemented group; on the other hand, Guangzhi Ning et al^[26]^ showed that cemented hemiarthroplasty did not reduce the risk of residual pain. Our pooled data from the meta-analysis comparing cemented with uncemented hemiarthroplasty suggested that the cemented group is associated with a long operation time. A previous meta-analysis^[26]^ reported the same results. However, Veldman et al^[27]^ reported that the mean operating time was 9 minutes shorter for cementless stems than for uncemented stems. Many potential factors, such as the type of prosthesis and doctor's skill, may affect this clinical outcome. In our study, we also compared the mortality and HHSs between the 2 groups at different times. Our findings showed that there was no statistically significant difference in mortality rate between the 2 groups in terms of short-term postoperative mortality and that the 1-year mortality in the cement group was lower than that in the uncemented group; Tao Li et al^[4]^ found that the use of cement did not increase the mortality 1 year postoperatively. Long-term mortality may better indicate the difference in mortality between the 2 groups and our finding showed that there was no statistically significant difference in mortality rates between the 2 groups at 2 and 4 years. Some studies^[29]^ also showed that there was no significant difference in the mortality rate between the 2 groups at the 12 month follow-up. Many risk factors, such as deteriorated preoperative cardiopulmonary function, old age, and physical reserve, may increase patient mortality.^[30,31]^ Regarding hip function, due to the various outcome parameters adopted for the assessment of postoperative hip function, it was difficult to pool all the results. Therefore, we compared the HHS at different times using 3 eligible RCTs. Our findings showed that the HHS at 3 months, 6 months, and 1 year were not significantly different between the cemented hemiarthroplasty groups and the uncemented hemiarthroplasty groups. However Vidovic et al^[21]^ supported the view that cemented hemiarthroplasty should be used for the management of displaced femoral neck fractures, as it provides better functional outcomes than uncemented hemiarthroplasty. However, in Langslet et al,^[11]^ a 5-year follow-up of a randomized trial showed that the HHSs at 5 years were higher in the uncemented group than in the cemented group (86.2 vs 76.3; mean difference 9.9; 95% CI, 1.9–17.9). Finally, we compared the incidence of complications between the 2 groups. In total, 9 of the included studies reported implant-related complications and the pooled results showed that cemented hemiarthroplasty has a lower risk of implant-related complications compared with uncemented hemiarthroplasty with small heterogeneity. Jameson et al^[32]^ reported that the uncemented group had more intraoperative and postoperative prosthesis loosening, periprosthetic fractures, and dislocation. Previous studies^[4,33–36]^ also concluded that cemented stems have fewer implant-related complications than cementless stems. Therefore, surgeons should pay attention to these possible implant-related complications before surgery. We also found no significant difference between the cemented group and the uncemented group in term of local complications and general complications. This suggests that cement has little, if not no, effect on local complications and general complications. It is worth considering that there is no difference in the rate of cardiovascular complications between the 2 groups. Some previous studies^[7,37,38]^ reported that the cement prosthesis may increase the risk of hypoxemia and transient hypotension, cardiovascular accidents, and pulmonary embolism. Therefore, high-quality evidence and well-designed RCTs are still necessary.

Compared with previous meta-analyses, there are some advantages to our study. First, we used an exhaustive search strategy and more strict inclusion criteria. A total of 15 newly published, high-quality RCTs were strictly included in this study to provide more effective evidence. Second, our study analyzed clinical outcomes including the length of hospital stay, operation time, reoperation rate, residual pain, blood loss, mortality, HHS, and complications. Mortality was further stratified into short-term postoperative mortality and mortality at 1 year, mortality at 2 years, and mortality at 4 years postoperatively. Complication were also divided into 4 subgroups: implant-related complications, cardiovascular complications, local complications, and general complication. It can reduce the potential bias risk from pooling all kinds of mortality and complications. Third, the HHS was used as to evaluate hip function to reduce the deviation of descriptive analysis.

However, our research still has some limitations. The limitations of this meta-analysis involve the restrictions on the publication language, the uniformity of the surgery administration programme and rehabilitation scheme, and the small sample size of the included studies. The distorting effects of location bias and publication bias on systematic reviews and meta-analyses are well documented.^[39–41]^ The variety of methods used to assess the functional results in the included studies, made it difficult to carry out a quantitative synthesis of the functional results. Due to certain features of the surgery techniques, it is impossible to blind orthopedic surgeons. Consequently, caution should be taken when interpreting the estimates of this meta-analysis. Finally, our evidence showed considerable statistical heterogeneity for several outcomes across the trials; however, the regression analysis and sensitivity analysis suggested that the results were stable.

## 5 Conclusions

Cemented hemiarthroplasty for elderly patients with displaced fracture of femoral neck may acquire better functional results.

## Author contributions

**Data curation:** Binfeng Liu, Ang Li.

**Formal analysis:** Jialin Wang, Hongbo Wang, Xiaoyu Lian.

**Investigation:** Gongwei Zhai, Haohao Ma, Bo Zhang.

**Methodology:** Binfeng Liu, Ang Li.

**Project administration:** Jialin Wang.

**Resources:** Yanzheng Gao, Liyun Liu.

**Software:** Liyun Liu.

**Validation:** Binfeng Liu.

**Writing – original draft:** Binfeng Liu.

**Writing – review & editing:** Yanzheng Gao.
